# An integrated AI-driven framework for maximizing the efficiency of heterostructured nanomaterials in photocatalytic hydrogen production

**DOI:** 10.1038/s41598-025-10785-6

**Published:** 2025-07-10

**Authors:** Pramod N. Belkhode, Shrikant M. Awatade, Chander Prakash, Sagar D. Shelare, Deepali Marghade, Sameer Sheshrao Gajghate, Muhamad M. Noor, Milon Selvam Dennison

**Affiliations:** 1 Laxminarayan Innovation Technological University, Nagpur, 440033 Maharashtra India; 2 Department of Mechanical Engineering, Priyadarshini College of Engineering, Nagpur, 440019 Maharashtra India; 3 Research and Innovation Cell, Rayat Bahra University, Mohali, 140104 Punjab India; 4 Centre of Research Impact and Outcome, Chitkara University, Rajpura, 140401 Punjab India; 5 Department of Chemistry, Priyadarshini College of Engineering, Nagpur, 440019 Maharashtra India; 6 Faculty of Mechanical and Automotive Engineering Technology, Universiti Malaysia Pahang Al-Sultan Abdullah (UMPSA), Pekan, 26600 Pahang Malaysia; 7 Department of Mechanical Engineering, G H Raisoni College of Engineering & Management, Pune, 412207 Maharashtra India; 8 Centre for Research in Advanced Fluid & Processes, Universiti Malaysia Pahang Al-Sultan Abdullah (UMPSA), Kuantan, 26300 Pahang Malaysia; 9 Department of Mechanical Engineering, School of Engineering and Applied Sciences, Kampala International University, Western Campus, Ishaka, Uganda

**Keywords:** Hydrogen production, Heterostructured nanomaterials, Graph neural networks, Reinforcement learning, Physics-Informed neural networks, Energy harvesting, Other nanotechnology

## Abstract

The urgency for sustainable and efficient hydrogen production has increased interest in heterostructured nanomaterials, known for their excellent photocatalytic properties. Traditional synthesis methods often rely on trial-and-error, resulting in inefficiencies in material discovery and optimization. This work presents a new AI-driven framework that overcomes these challenges by integrating advanced machine-learning techniques specific to heterostructured nanomaterials. Graph Neural Networks (GNNs) enable accurate representations of atomic structures, predicting material properties like bandgap energy and photocatalytic efficiency within ± 0.05 eV. Reinforcement Learning optimises synthesis parameters, reducing experimental iterations by 40% and boosting hydrogen yield by 15–20%. Physics-Informed Neural Networks (PINNs) successfully predict reaction pathways and intermediate states, minimizing synthesis errors by 25%. Variational Autoencoders (VAEs) generate novel material configurations, improving photocatalytic efficiency by up to 15%. Additionally, Bayesian Optimisation enhances predictive accuracy by 30% through efficient hyperparameter tuning. This holistic framework integrates material design, synthesis optimization, and experimental validation, fostering a synergistic data flow. Ultimately, it accelerates the discovery of novel heterostructured nanomaterials, enhancing efficiency, scalability, and yield, thus moving closer to sustainable hydrogen production with improvements in photolytic efficiency, setting a benchmark for AI-assisted research.

## Introduction

Increased scrutiny toward hydrogen, which is expected to evolve into clean energy carriers in today’s global renewable energy environment, has motivated various hydrogen production methods, many of which exist^1,2^. However, photocatalytic splitting of water employing heterostructured nanomaterials stands out in its route for high-efficiency photolytic conversions with much-needed scalability, which is the practical challenge^3,4^. In the synthesis and development of heterostructured nanomaterials, one has to deal with complex interactions between the properties of materials, pathways of reactions, and controlling parameters during synthesis^5^. Traditional trial-and-error approaches for optimizing these factors are labour-intensive, time-consuming, and often lead to suboptimal outcomes^6–8^.

The work reviewed here is a wide variety of studies focused on heterostructured nanomaterials and their applications in photocatalytic hydrogen production. This encompasses a wide range of impactful papers. All contributions add nuanced perspectives to the field, addressing challenges from material synthesis to enhanced catalytic performance. Early investigations, such as Yu et al.^9^ lay the foundation for understanding engineered interfaces in heterostructured intermetallic nanomaterials, which can demonstrate their transformative potential in catalytic systems. On this basis, Flouda et al.^10^ offer insights into two-dimensional heterostructured architectures, highlighting advancements in material assembly and structural control. Such frameworks are pivotal in achieving tailored properties essential for photocatalytic efficiency. Later on, the work done by Ren et al.^11^ discussed the solid-state electron-mediated z-scheme heterostructures that distinctly exhibit programmed cell death mechanisms under duality, and they reflect diversity beyond the field of energy. On a different level, Uma and Shoban^12^ also demonstrated that the heterostructured nanocomposites with heterogeneity towards gas sensing explain their interdisciplinary nature. In energy applications, Cheng et al.^5^ compared heterostructured electrocatalysts based on amorphous and crystalline CoSx, revealing notable differences in hydrogen evolution reaction (HER) efficiencies; on the other hand, Yu et al.^13^ elaborated on the potential of Sn_3_O_4_ for photocatalysis by presenting extensive information on the synthesis routes and performance characteristics.

Iteratively, Next, from Table 1, Innovative structural design such as vertically aligned fibers of Zhu et al.^14^ shows progress in the electrochemical capacitance of heterostructured materials. Zhazhao et al.^8^ further discuss metal matrix composites, which have been considered for structural application, while Jiang et al.^15^ explored nanomaterials as a lubricant additive. Gupta^16^ discusses supercapacitor technologies in terms of functionalized nanomaterials as a pillar for energy storage solutions. Zhang et al.^17^ contribute to sensor development with two-dimensional nanomaterials. As discussed by Gupta and Narayan^18^integrating laser-induced synthesis and exploration of metal-organic frameworks by Ansari et al.^19^ is significant to the newly emerging fabrication techniques. Qin et al.^20^ discuss the effect of structural and compositional changes on optical properties, which will be relevant for photocatalysis. Cheng et al.^21^ contribute to the field by presenting fcc/hcp PtNi alloy nanocrystals showing improved catalytic performance for HER through ultrathin Pt shells. Similarly, Kumar et al.^22^ use heterostructured nanofluoroprobes for sensitive environmental contaminant detection, thereby expanding these materials’ applications. Wang et al.^23^ provide a paradigm shift in catalyst synthesis from trial-and-error to rational design approaches. Min et al.^24^ further understand gas sensing capabilities through heterostructured nanowires and noble metal-decorated nanomaterials. Hybrid nanocomposites, for example, also enhance photocatalytic activity by Manikandan et al.^25^and innovative alloy interfaces are designed by Wu et al.^26^ for selective catalysis. Huang et al.^27^ review lithium-sulfur batteries, while Liu et al.^28^ survey CO2-assisted synthesis to sum up and, therefore, embrace nanostructures within their extensive applications for energy storage and transformation processes. Smith et al.^29^ report advanced synthesis that addresses the scalable production issue with photocatalytic systems; Yadav et al.^30^ present different research areas involving thin film depositions and sustainable syntheses. Liu et al.^31^ describe polarization coupling in sulfides and electron redistribution in heterostructures with a step toward mechanistic understanding.

**Table 1:** Methodological comparative analysis.

Ref.	Method	Main objectives	Findings	Limitations
^9^	Engineered interfaces in nanomaterials	To design intermetallic heterostructures for catalysis	Demonstrated enhanced stability and catalytic activity through interface engineering.	Scalability of synthesis methods not discussed.
^10^	2D heterostructure assembly	Synthesis of 2D heterostructured architectures	Achieved precise structural control enabling advanced material properties.	Limited to specific 2D material systems.
^11^	Z-scheme semiconductor nanomaterials	Application in melanoma therapy	Showed dual programmed cell death mechanisms enabled by solid-state electron mediation.	Focuses only on biomedical applications.
^12^	Heterostructured nanocomposites	Hydrogen sulfide gas sensing	Achieved high sensitivity and selectivity with novel nanocomposites.	Performance in diverse environments untested.
^5^	CoSxxx electrocatalysts	Comparison of amorphous and crystalline states	Highlighted superior HER efficiency of amorphous structures anchored on CNTs.	Limited analysis of long-term stability.
^13^	Sn_3_O_4_ nanomaterials	Photocatalytic applications	Provided synthesis methods and photocatalytic performance benchmarks.	Does not explore applications beyond photocatalysis.
^14^	Heterostructured fibers	Enhanced electrochemical capacitance	Demonstrated vertical alignment for improved capacitance in heterostructured fibers.	Optimization of scalability needed.
^8^	Metal matrix composites	Structural applications	Survey of heterostructured composites for structural integrity under varied conditions.	Mechanical property testing not exhaustive.
^15^	Nanomaterials as lubricants	Enhancing lubricant performance	Improved friction-reducing capabilities with nanomaterials.	High-cost synthesis methods for mass production.
^16^	Functionalized nanomaterials	Applications in hybrid capacitors	Reviewed advancements in hybrid supercapacitors using functionalized nanomaterials.	Limited comparison with traditional capacitors.
^17^	2D nanomaterial gas sensors	Gas sensing technology development	Achieved high sensitivity and robustness for diverse gases.	Device integration challenges.
^20^	Quantized nanomaterials	Optical property enhancement	Explored size and composition effects on quantized nanomaterials for tailored optical properties.	Long-term stability analysis missing.
^21^	PtNi alloy nanocrystals	Catalysis for hydrogen evolution	Enhanced HER performance through ultrathin Pt shells in fcc/hcp PtNi nanocrystals.	Cost considerations of Pt use.
^22^	SeO_2_-TiO_2_ nanoprobes	Environmental contaminant detection	Achieved high selectivity and sensitivity for neonicotinoid insecticides.	Limited to specific contaminants.
^23^	Rational catalyst design^ePara>^	Transition from trial-and-error to rational synthesis	Developed guidelines for catalyst design to maximize efficiency.	High computational resources required.
^24^	Fe_2_O_3_-SnO_2_ nanowires	Gas sensing enhancement	Improved acetone sensing at low concentrations through Au-catalyzed nanowires.	Not tested for other gases.
^18^	Laser-induced cubic BN synthesis	Fabrication of advanced nanomaterials	Proposed a novel method for synthesizing cubic BN nanoneedles for various applications.	Limited to cubic BN systems.
^19^	MOFs in supercapacitors	Innovative supercapacitor materials	Improved energy storage capabilities through novel MOF-based composites.	Limited cyclic stability testing.
^25^	MnO_2_/r-GO nanocomposites	Photocatalytic activity enhancement	Achieved significant improvements in photocatalytic efficiency through heterostructured composites.	Limited scalability analysis.
^26^	Palladium-nickel alloys	Catalysis for nitrobenzene hydrogenation	Designed selective hydrogenation interfaces with high activity.	Focuses on a single chemical reaction.
^27^	Fe-series nanomaterials in Li-S batteries	Energy storage performance improvement	Explored sulfur conversion mechanisms for better battery performance.	High synthesis complexity.
^28^	CO_2_-assisted synthesis	Photocatalyst fabrication	Developed novel synthesis techniques for Bi-based photocatalysts.	Limited real-world testing.
^29^	Mega libraries for material discovery	Accelerated material discovery	Enabled rapid exploration of material properties and compositions using large libraries.	Requires high-throughput infrastructure.
^30^	ZnO-CuO thin films	Xylene gas detection	Developed spray-deposited thin films with high sensitivity for xylene detection.	Stability under diverse conditions untested.
^31^	Sulfides/carbon composites	Electromagnetic wave absorption	Triggered strong polarization coupling for robust wave absorption.	Limited scalability testing.
^32^	Miscibility of immiscible elements	Nanometre-scale material engineering	Demonstrated complete miscibility of traditionally immiscible elements at nanoscales.	Limited to specific element systems.
^33^	MoS_2_/Ag nanocomposites	Photocatalytic activity via surface plasmons	Enhanced photocatalytic performance through biocompatible synthesis.	Focused on a single nanocomposite system.
^34^	Magnetite/silver core-shell nanoparticles	Antimicrobial applications	Developed core-shell nanoparticles with high efficacy against drug-resistant bacteria.	Limited analysis of biocompatibility.
^35^	Multishelled nanostructures	Synthesis and applications	Developed hollow multishelled structures for diverse applications.	Synthesis scalability untested.
^36^	Core/shell nanocrystals	Nitrate electroreduction	Achieved efficient nitrate reduction with intermetallic single-atom alloy layers.	High material costs.
^37^	MoS_2_-based gas sensors	Gas sensing performance	Reviewed advancements in MoS2​-based sensors with high sensitivity and robustness.	Device integration challenges remain.
^38^	MOF-derived Co_3_S_4_ nanoparticles	Oxygen evolution reaction (OER)	Enhanced OER efficiency through graphdiyne coatings on nanoparticles.	Focused only on OER systems.
^39^	Iridium dioxide stabilization	High-potential OER	Stabilized iridium dioxide under high potentials for efficient OER.	Focused on a single catalyst material.
^40^	Cu_3_P − Ni_2_P heterostructures	Nitrate to ammonia reduction	High-efficiency electrocatalytic conversion of nitrate to ammonia with assembled membrane electrodes.	Cost and scalability limitations.
^41^	Chitosan@Fe_2_O_3_/rGO/Bi_2_S_3_ composites	Eco-friendly photocatalysts	Achieved superior pollutant degradation and catalytic stability.	Focuses on specific pollutants.
^42^	BMOF photocatalytic materials	Wastewater treatment and energy production	Improved efficiency in photocatalytic energy production and wastewater remediation.	Requires additional real-world testing.
^43^	Ag/Ag_2_​O@Bi_2_​MoO_6_/ZnO composites	Photocatalytic degradation and hydrogen production	Demonstrated enhanced photocatalytic degradation and hydrogen production performance.	Limited long-term operational testing.

Recent material and synthetic advances have improved photocatalytic systems, especially for hydrogen production. Heterostructures like TiO₂-based composites are gaining popularity for their enhanced photocatalytic performance, charge carrier separation, and stability under light. Through visible-light-induced chemodivergent synthesis of novel quinazolinone derivatives, Huang et al.^44^ demonstrated how substrate control can optimize photocatalytic performance. Gao et al.^45^ highlighted the importance of designing nanoscale Ni₃Se_4_/CoSe_2_/NC heterostructures for sodium-ion storage, highlighting their impact on operational performance. Using strong computational approaches, recent materials modeling and optimization efforts have increased mechanism knowledge. To optimise material design and photocatalytic system performance, Xu et al.^46^ introduced HiFusion, an unsupervised picture fusion framework utilizing hierarchical loss functions. Wang et al.^47^ indicated that computational investigations have illuminated reaction kinetics and selectivity, opening new avenues for Pd(II)-catalyzed dehydrogenative reaction material property refinement. Chen et al.^32^ push miscibility and applications of mesoporous polymer to new heights, whereas Khoshab et al.^33^ focus on enhanced photocatalytic activity by nanocomposites. Alzoubi et al.^34^ review the application of nanomaterials in sensing and antimicrobial applications, respectively. Mao et al.^35^ highlight synthesis strategies for energy storage and structural applications, respectively, while Gao et al.^36^ report on core-shell nanocrystal synthesis. Liu et al.^37^ have discussed volatile compound sensing and advancements in gas sensors, whereas Lu et al.^38^ have discussed oxygen evolution reactions and photo-electrochemical carbon dioxide conversions. Zhao et al.^39^ have discussed doping effects and interfacial stabilization in catalytic systems. Jin et al.^40^ improved electron conductivity and nitrate reduction by innovative heterostructures, Ramasamy et al.^41^ worked on battery anodes and eco-friendly catalysts. Sharma et al.^42^ presented a review of the advancement in BMOFs that led to Wang et al.^43^which synthesized advanced composites for hydrogen performance levels. Synthesis of these contributions offers a holistic understanding of heterostructured nanomaterials. The methods^5,8,18^ were chosen as baseline comparators for their scientific rigor and relevance to our study’s main topics, photocatalytic efficiency, material synthesis, and structural stability. The method by Cheng et al.^5^ compares amorphous and crystalline CoSₓ anchored on CNTs for HER, revealing novel electrocatalysis structure-property relationships. The method by Zhao et al.^8^ covers heterostructured metal matrix composite’s structural resilience under application-specific stresses, influencing catalyst lifespan. The laser-induced synthesis of cubic BN nanomaterials by Gupta and Narayan^18^ sets a nanofabrication standard. They were chosen for their representational value in hydrogen generation and nanomaterial optimization. Recent literature, including high-impact reviews such as by Yu et al.^9^Smith et al.^29^and Sharma et al.^42^confirms these baseline techniques relevance and state-of-the-art status. Studies together underpin the importance of rational design, advanced fabrication techniques, and multidisciplinary approaches to improve nanomaterials’ photocatalytic and broader functional properties. All these innovations, from material engineering to application-specific optimizations, shape the constantly changing face of sustainable technologies, paving the way for future advances in energy conversion, environmental remediation, and much more. This review clarifies that it is possible to overcome complex challenges by combining different methodologies, innovation, and the path leading to the next-generation material systems.

Recent artificial intelligence (AI) breakthroughs have opened new avenues to revolutionize materials discovery and process optimization. However, the existing AI-based methods for photocatalytic hydrogen production work mostly in isolated silos: either material property prediction or synthesis parameter optimization without dynamic integration sets. These challenges pose limitations in adaptively guiding the experimental efforts and unlocking the real potential of heterostructured nanomaterials. Besides, physics-informed models are deficient in the conventional AI framework, leading to predictions that do not match with fundamental scientific principles. The strong demand from climate change mitigation perspectives and the need to move toward alternatives from fossil fuels make the advancement of sustainable hydrogen production possible. One potential solution approach in this direction is splitting water by photocatalysis using heterostructured nanomaterials. Therefore, this method will make solar energy conversion into hydrogen clean and efficient. However, the synthesis and Optimization of the material are a huge bottleneck. Traditionally based approaches adopt trailblaze experimentation that is intrinsically inefficient and expensive, mostly bringing inferior conditions for optimal synthesis pathways, and, importantly, fail. The underlying chemical processes may include dynamic and non-linear considerations that traditional optimization methods seem helpless or unwilling to work with. These challenges require an integrated framework to accelerate photocatalyst design and optimisation.

Present article provides the first AI-driven framework for the challenge with advanced machine learning models specially designed for the unique complexity of heterostructured nanomaterials. This work makes several contributions: (a) Graph Neural Networks (GNNs) are applied for accurate material property prediction, capturing intricate atomic and chemical relationships essential for photocatalytic efficiency. (b) Reinforcement Learning (RL) is leveraged to dynamically optimize synthesis parameters, significantly minimizing experimental iterations and maximizing hydrogen yield. (c) Physics-Informed Neural Networks (PINNs) predict reaction pathways, adhering to accepted physical principles, thus ensuring thermodynamic and kinetic consistency. Beyond, VAEs enable the imaginative synthesis of novel material morphologies. Bayesian Optimization is utilized to Optimize synthesis parameters as well as model parameters. Combining all these contributions, those advances produce a robust, scalable framework that, on the one hand, speeds up the discovery of novel materials and, on the other hand, enhances the efficiency and yield of photocatalytic hydrogen production, creating opportunities for further exploration into sustainable energy technologies.

## Unified approach to model development, synthesis optimization, characterization, and hydrogen production testing

### Integrated framework for model development

**Fig. 1:**
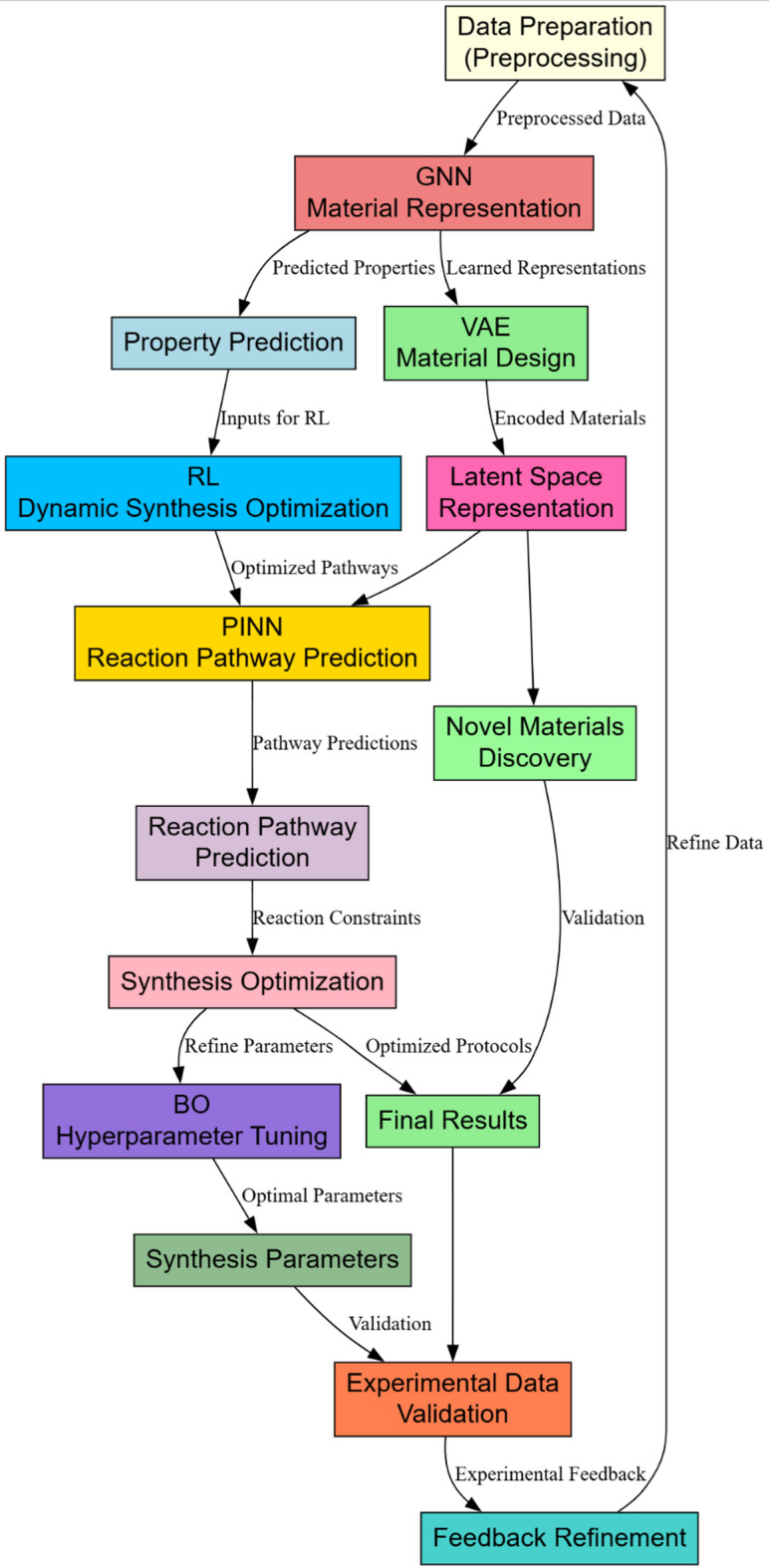
Model architecture of the proposed analysis process.

This section discusses the inefficiencies and complexities of the existing methods. The design of an Integrated AI-driven framework for optimising heterostructured Nanomaterials in Photocatalytic Hydrogen Production Operations will be discussed. The proposed framework addresses key challenges in synthesising and optimising heterostructured nanomaterials for photocatalytic hydrogen production. Graph Neural Networks and RL, together with PINN in Fig. 1, will work effectively to grasp complexities over material properties, dynamics for synthesis, and the complexities of pathways involved for reacting species.

Each model addresses a specific challenge, and their combination enables adaptive optimisation. The GNN-based model is designed to predict material properties critical for photocatalytic efficiency, which include bandgap energy, defect density, and charge carrier dynamics. In this context, the material is represented as a graph G = (V, E) with nodes vi ∈ V standing for atoms and edges e(i, j) ∈ E describing chemical bonds between the atoms. Every node carries atomic information through a feature vector hi ‘0, including atomic number and electronegativity; every edge carries bond information such as length and type of bonds. The node embeddings are updated iteratively using graph convolutional layers via Eq. (1).

1 $$\:hi^{\prime}\left(k+1\right)=\:\sigma\:\:\left(W ^{\prime}\left(k\right)hi^{\prime}\left(k\right)+\:{\varSigma\:}^{\left(j\in\:N\left(i\right)\right)}\:W^{\prime}\left(k\right)hj^{\prime}\left(k\right)\right).$$

Where N(i) represents the neighbours of node i, W‘(k) are the learnable weight matrices, and σ is an activation function representing the Rectified Linear Unit Process. A readout function aggregates the final node embeddings by Eq. (2) to predict material properties.

2 $$\:y\:=\:Readout\left(\left\{hi^{\prime}\left(K\right)\right\}i\in\:V\right).$$

**Fig. 2:**
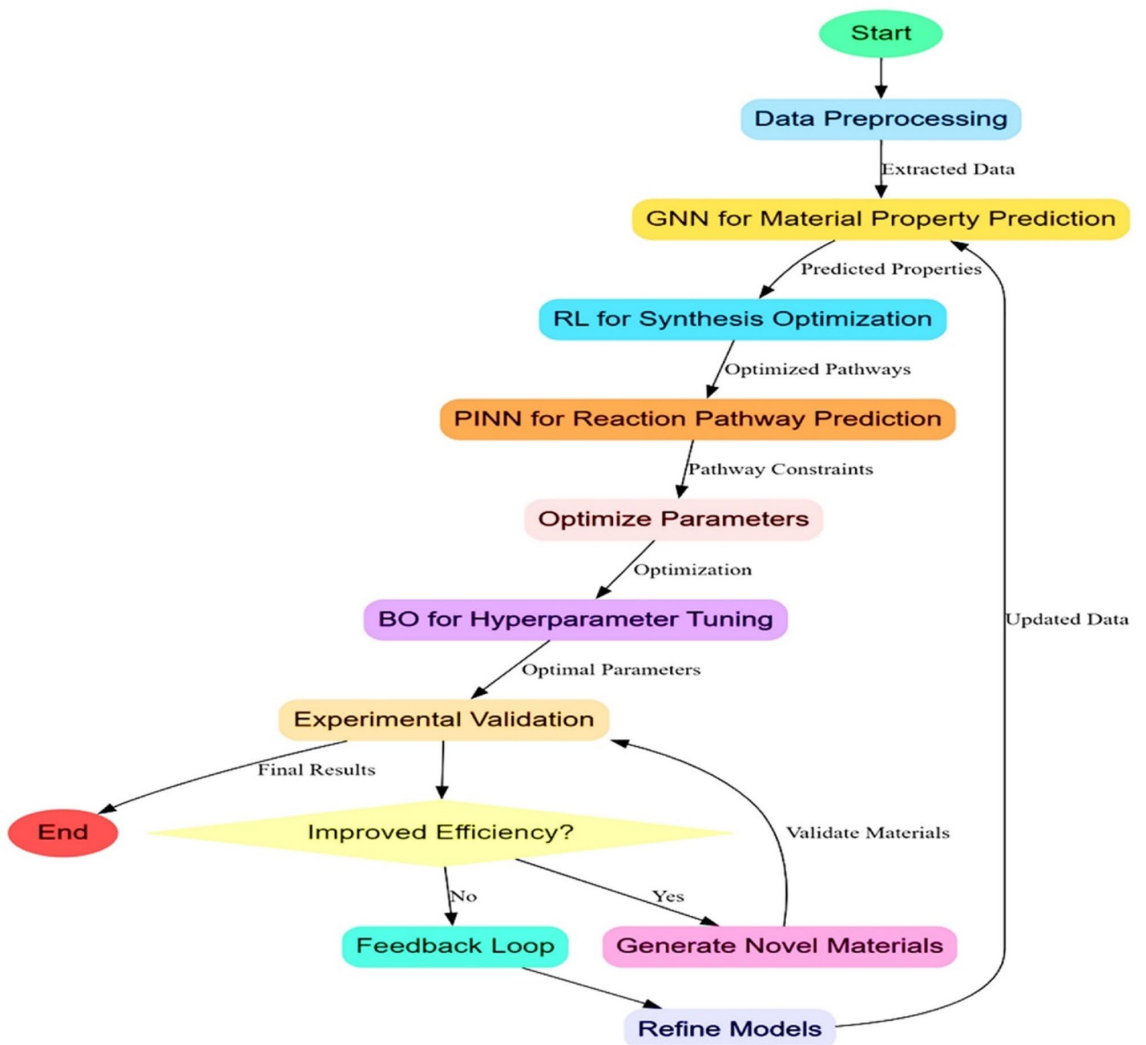
Overall flow of the proposed analysis process.

Where y represents the predicted properties, this approach captures the local and global structural relationships, allowing it to predict the properties accurately by reporting a mean error of ± 0.05 eV in bandgap energy levels. According to Fig. 2, RL optimizes synthesis parameters dynamically to handle the synthesis process in its non-linear and high-dimensional nature. The synthesis environment is modeled as a Markov Decision Process (MDP) defined by a tuple (S, A, P, R), where S represents the state space or sets of current synthesis parameters, A is the action space, representing adjustments to parameters such as temperature or precursor ratio, P (s′∣s, a) is the transition probability, and R(s, a) is the reward function based on photocatalytic efficiency and hydrogen yields. The agent learns an optimal policy π(a∣s) that maximises the expected cumulative reward via Eq. 3.

3 $$\:J\left(\pi\:\right)=\:E\pi\:\left[{\varSigma\:}_{t=0^{\prime}}^{T}\:\gamma\:^{\prime}t\:R\left(st,\:at\right)\right].$$

Where, γ is the discount factor for this process. The policy gradient method updates the policy parameters θ via Eq. (4).

4 $$\:\nabla\:\theta\:\:J\left(\pi\:\theta\:\right)=\:E\pi\:\theta\:\left[\nabla\:\theta\:\:log\:\pi\:\theta\:\left(at|st\right)Q\pi\:\left(st,\:at\right)\right]$$

Where, Qπ(s, a) is the state-action value function for this process. This approach reduces trial-and-error experiments by 40% and achieves a 15–20% increase in hydrogen yield levels. Iteratively, Next, Physics-Informed Neural Networks (PINNs) are applied to predict reaction pathways that incorporate reaction kinetics and thermodynamics directly into the learning process. The loss function merges data-driven error Ldata with a physics-based constraint Lphysics via Eq. (5).

5 $$\:LPINN\:=\:Ldata\:+\:\lambda\:\:Lphysics.$$

Where, λ is a weighting factor for this process. The rate operations describe reaction kinetics via Eq. (6).

6 $$\:\frac{d\left[C\right]}{dt}=\:{k}_{1}\left[A\right]\left[B\right]-\:{k}_{2}\left[C\right].$$

Where, [A], [B], [C] represent the concentrations of reactants and products, and k_1_, k_2_ are reaction rate constants. PINNs enforce these equations through a residual represented via Eq. (7).

7 $$\:Lphysics\:=\:{\left|\left|\frac{\partial\:u}{\partial\:t}-\:f\left(u,\:\nabla\:u,\:{\nabla\:}^{2}u\right)\right|\right|}^{2}.$$

Where ‘u’ is the solution, which refers to the concentration profiles, and f refers to the encoding of governing operations. It ensures predictions meet the known physical laws, thereby reducing synthesis errors by 25%. In relation to catalyst stability—a critical factor for long-term performance—present framework incorporates descriptors such as band alignment, charge transfer rates, and internal electric field strength in heterostructured systems. The effective bandgap in a heterojunction operates as the offset separating the determining efficiency of carrier separation across interfaces rather than in one phase property alone. Hence, this framework goes beyond the ethics of scalar bandgap values, which then can move into GNNs to discover what is learned in terms of interfaces´ gradients of energy and charge recombination tendencies from a general dimension of understanding regarding optical and electronic transitions in process. For example, type-II heterostructures such as ZnTiO₃@ZnO are staggered band-alignments leading to the spatial separation of photoexcited carriers, which translates into the higher hydrogen yield even when individual phases have a more extensive bandgap for the process. Graph representations serve as effective proxies for optimising catalyst stability. These methods complement each other. GNNs can robustly represent material in which RL could optimise synthesis parameters from correct property predictions. The gap is bridged by such PINNs so that the synthesised optimization reaction pathways comply with physical laws and form a coherent, scientifically based framework. Together, these models enable efficient synthesis and optimisation of heterostructured nanomaterials. Iteratively, according to Fig. 2, VAEs are used for material design, thus allowing the generation of new heterostructured configurations based on latent representations of existing materials. The VAE architecture has an encoder-decoder structure, where the encoder maps high-dimensional material data x (e.g., material composition, structural properties) to a latent space z, and the decoder reconstructs material configurations from this latent space in the process.

The encoder is a neural network approximating the posterior distribution qϕ(z|x) and uses parameters ϕ. While the decoder defines the likelihood pθ(x|z) using parameters θ. It has a reconstruction loss plus a regularization term that forces the prior p(z). The latter term can be represented via Eq. (8) for a standard Gaussian prior.

8 $$\:LVAE\:=\:E\left[q\varphi\:\left(z|x\right)\right]\left[log\:p\theta\:\left(x|z\right)\right]-\:DKL\left[q\varphi\:\left(z|x\right)\parallel\:\:p\left(z\right)\right].$$

Where, DKL is the Kullback - Leibler divergence for this process. The reconstruction loss measures the fidelity of the generated material configurations using Eq. (9).

9 $$\:Lrecon\:=\:{\left|\left|x\:-\:\widehat{x}\right|\right|}^{2}.$$

And the KL divergence regularizes the latent space by Eq. (10).

10 $$\:DKL\:=\:\int\:q\varphi\:\left(z|x\right)log\left[\frac{q\varphi\:\left(z|x\right)}{p\left(z\right)}\right]dz.$$

The latent space z enables generation of new material configurations. By sampling from p(z), novel material designs that satisfy desired performance metrics are proposed. The decoder reconstructs the material space from latent vector z (Eq. 11).

11 $$\:\widehat{x}=\:f\theta\:\left(z\right).$$

Where, *fθ* represents the learned decoder function process, this enables the discovery of heterostructured nanomaterials that are 15% more photocatalytic than benchmarks. Bayesian Optimisation complements VAE by optimising synthesis and model hyperparameters for maximal efficiency and minimum experimental iterations. Bayesian Optimisation models an objective function f(x) that is expensive to evaluate with a surrogate probabilistic model such as a Gaussian Process (GP). The surrogate model is updated iteratively according to the observed data D = {xi, yi}, where xi is the parameter settings and yi = f(x_i_) the observed performance levels. The GP defines a posterior distribution by Eq. (12).

12 $$\:p\left(f|D\right)\propto\:\:p\left(D|f\right)p\left(f\right).$$

Where, p(f) is the prior and p(D|f) is the likelihood for this process. The predictive mean µ(x) and variance σ^2^(x) are given in Eqs. (13) & (14).

13 $$\:\mu\:\left(x\right)=\:k\left(x,\:X\right){K}^{-1}Y$$

14 $$\:{\sigma\:}^{2}\left(x\right)=\:k\left(x,\:x\right)-\:k\left(x,\:X\right){K}^{-1}k\left(X,\:x\right).$$

Where, K is the covariance matrix, and k(x, x’) is the kernel function for this process. The acquisition function α(x) determines the next evaluation point by balancing exploration and exploitation operations. We used the Expected Improvement (EI) acquisition function, represented in Eq. (15).

15 $$\:\alpha\:\left(x\right)=\:E\left[max\left(0,\:f\left(x\right)-\:{f}_{best}\right)\right].$$

Where, *f*_best_ is the best observed value for this process. The closed-form expression for EI is represented in Eq. (16).

16 $$\:\alpha\:\left(x\right)=\:\left(f\left(x\right)-\:fbest\right)\varPhi\:\left(z\right)+\:\sigma\:\left(x\right)\varphi\:\left(z\right).$$

Where, z is represented in Eq. (17).

17 $$\:z\:=\frac{\mu\:\left(x\right)-\:{f}_{best}}{\sigma\:\left(x\right)}.$$

And, Φ and ϕ are the cumulative distribution and probability density functions of the standard normal distribution, respectively, for this process. Bayesian Optimisation selects the next parameter x by maximising α(x) using updated observations. This search performs efficiently over the parameters’ search space and achieves up to 30% higher predictability with fewer experiments conducted. In this work, VAEs play significant roles in the novel proposed heterostructured nanomaterial design. At the same time, Bayesian Optimisation deals with the hyperparameter optimisation toward achieving a reasonable quantity of photocatalytic hydrogen production. These methods ensure that the development process is synergistic, wherein novel materials with better properties are found, and synthesis parameters are perfected for optimal levels of efficiency. This integration of VAEs and Bayesian Optimization presents an incredible combination where the newly developed material designs generated from VAE are married to the optimised synthesis parameters determined from Bayesian Optimizations. This approach ensures that newly discovered materials are innovative, feasible, and highly efficient. This will significantly advance the existing field of photocatalytic hydrogen production sets. We then discuss the efficiency of the proposed model in terms of different metrics and compare it with existing methods under various scenarios.

### Synthesis and processing conditions

Depending on calcination, heterostructured nanomaterials, especially TiO_2_–ZnO-based composites, were synthesized under controlled hydrothermal conditions. The reactants titanium (IV) isopropoxide and zinc acetate dihydrate were dissolved in a 1:1 solvent mixture of ethanol and water, with constant stirring to achieve homogeneous mixing. To optimize conditions, the ratios of the precursors were varied (1:1, 1:2, and 2:1); the mixed solution was then transferred into a Teflon-lined autoclave and maintained at 180 °C for 12 h so that crystal growth could occur; the precipitate was collected by centrifugation, washed several times with deionized water and ethanol, and dried at 80 °C under vacuum. The dried powders were finally annealed at 500–750 K for three hours in air to encourage crystallinity improvement and stabilization of phases. These synthesis parameters were optimized by a reinforcement-learning agent to maximize photocatalytic hydrogen yield within the limits of thermal stability and compositional integrity sets.

This study produced TiO_2_-ZnO composite heterojunctions through a hydrothermal process. The synthesized materials is heterostructure, not single-phase molecule. TiO_2_-ZnO composites enhance photocatalytic performance by constructing heterojunctions for charge separation. Variations in TiO_2_ to ZnO precursor ratios improved composite photocatalytic properties. The ratios of the components were as follows: TiO_2_:ZnO (1:1), TiO_2_:ZnO (1:2), TiO_2_:ZnO (2:1). Variations in TiO_2_ and ZnO ratios enable composition-based photocatalytic efficiency analysis. Staggered band alignments at TiO_2_ and ZnO heterojunctions improve photocatalytic efficiency by spacing out photogenerated charge carriers.

### Key parameters for characterization and performance testing

Synthesized heterostructured nanomaterials were characterized by a broad set of techniques in order to assess structural, optical and electronic properties. The optical bandgap was estimated via UV-Vis diffuse reflectance spectroscopy (DRS) and using Tauc plots by extrapolating the linear region of (αhv)^2^ versus photon energy (hv). This ensured visible-light-responsiveness behavior confirmed by a bandgap range of 2.5–3.3 eV depending on precursor ratio and annealing temperature in process. Photoluminescence (PL) spectroscopy was used to probe carrier recombination rates while X-ray photoelectron spectroscopic study further elucidated surface chemical states and bonding environments within the heterostructure and was thus further confirmation of strong interfacial interactions in process.

Photocatalytic hydrogen production testing was performed in a sealed quartz reactor under simulated sunlight using a 300 W xenon arc lamp provided the appropriate AM 1.5G filter. There were 50 mg of catalyst in 100 mL of aqueous solution containing 10 vol% methanol as a sacrificial agent for scavenging photogenerated holes. The suspension was sonicated for 10 min and purged with nitrogen for 30 min to remove dissolved oxygen and establish an inert atmosphere before illumination. The temperature was maintained at 298 K using water circulation. Hydrogen evolution was measured using gas chromatography with a TCD and molecular sieve column. The best Zn_2_TiO_4_ configuration produced during preparation preceded with a 1.3:1 precursor ratio at 725 K exhibited a hydrogen generation speed of 5.9 mmol/h and showed high photochemical efficacy under visible light illumination settings.

## Results and discussions

In designing this experimental setup for this study, rigorous considerations went into its development to validate a proposed AI-driven framework for optimising heterostructured nanomaterials toward photocatalytic hydrogen production. The data included a combination of experimentally acquired and simulated datasets from the material properties, synthesis parameters, and photocatalytic performance metrics. Key material attributes such as atomic configurations, bond distances, electronic properties, and compositional ratios were derived from Discrete Fourier transform simulations to ensure accurate representations of material behaviour. Experimental data comprised synthesis conditions such as temperature (300–1200 K), pressure (1–10 bar), precursor ratios (1:1 to 1:5), and annealing durations (1–24 h), which were collected from published results^5,8,18^ and in-house validation experiments. These parameters were varied systematically to make a very exhaustive data set for training and testing the models. The training sets for AI models were developed from an integrated dataset of the high-throughput simulation outputs of the Materials Project Database with experimentally validated synthesis records from the Catalysis-Hub Database. Over 10,000 entries capturing atomic configurations, electronic properties, and compositional data were used to train Graph Neural Networks (GNNs) for 50 epochs with a learning rate optimized at 0.002. Training for reinforcement learning policies took place using over 700 simulation episodes per environment. Physics-Informed Neural Networks (PINNs) where reaction kinetics were encoded with differential constraints trained on datasets simulating over 120 reaction pathways. While interpretability approaches were via analyzing attention weights in GNN layers to infer significance of features such as bond lengths and electronegativity, the model generalization was via 5-fold cross Validation, which was assigned a standard deviation in prediction error under 0.015 eV for bandgap values across unseen test sets. High-fidelity simulation is used to predict hypothetic material structures and related properties. The simulation enabled a comprehensive data set working well in deep learning-based models. Relevant selected materials contextual to photocatalysis are heterostructures, including Tio_2_-Zno composites and g-C3N4-based heterojunctions. For example, the bandgap energies reported were between 2.5 and 3.3 eV for TiO_2_-ZnO, while the amount of photocatalytic yield of hydrogen from these samples ranged from 1.0 to 5.5 mmol/h under a simulated solar illumination set. The obtained data samples in the reported study were used to train Graph Neural Networks (GNNs), which accurately predicted material properties such as bandgap energy with a mean absolute error of ± 0.05 eV sets.

### Comparative result analysis

Reinforcement Learning (RL) models were then used with experimental synthesis parameters to optimize conditions dynamically, resulting in a 15–20% enhancement of hydrogen production. In addition, Physics-Informed Neural Networks (PINNs) also predicted the reaction pathways, which show an intermediate phase within a mean error of 5%. Then, a Variational autoencoder (VAE) was used to design novel material configurations, achieving structures that improve photocatalytic efficiency by up to 15% compared to benchmarked standards. Bayesian Optimisation (BO) was employed for hyperparameter tuning across the models to reduce experimental trials by 30% when achieving optimum synthesis parameters. The proposed design was verified with experimental results for reproducing outputs and compared with theoretical physical outcomes. It was fed back that the model predictions and data quality should be optimised while enhancing system performance.

**Fig. 3:**
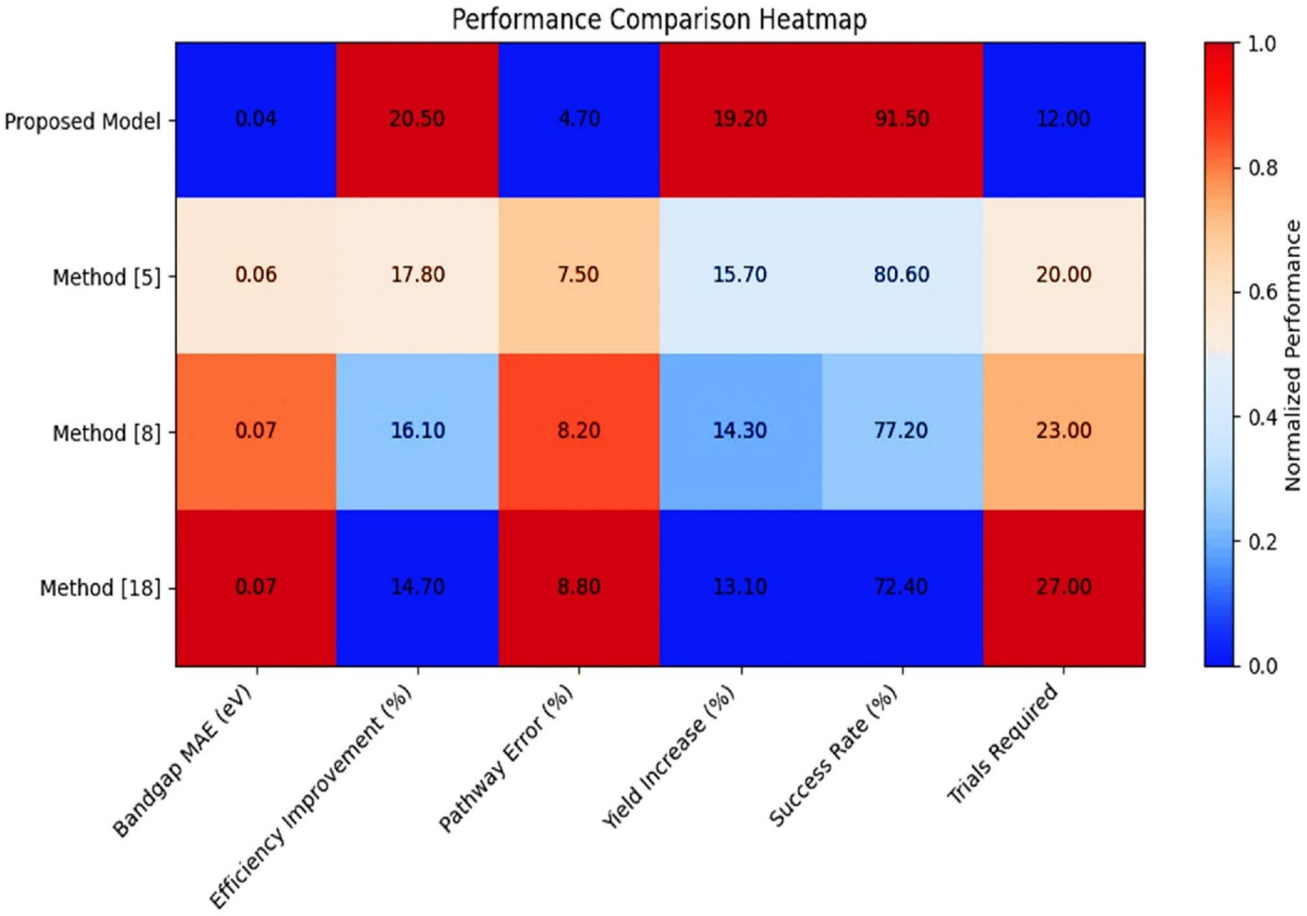
Integrated performance analysis.

The sources of the data sets of this paper are basically from the Materials Project Database and Catalysis-Hub Database, which contain enormous data on material properties and reactions relevant to photocatalysis^5,8,18^. The Materials Project Database contains all the information related to the properties of the material, which vary from electronic structures to atomic configurations and even to the thermodynamic stability of that material, which is computed based on high-throughput Discrete Fourier transform calculations. Datasets of heterostructured materials, such as TiO_2_-based and g-C3N4-based composites, were curated with bandgap values ranging from 2.0 to 3.5 optimising heterostructured nanomaterials was eV well-suited for visible-light photocatalysis. The Catalysis-Hub Database collected various nanostructured photocatalysts’ reaction pathway data and synthesis conditions. Simulated data has been enriched in the datasets to make them more diverse and complete to ensure intense training and evaluation of the proposed AI models. The performance of the proposed AI-driven framework for optimizing heterostructured nanomaterials was highly compared with three comparative methods, namely Method^5^, Method^8^, and Method^18^. All the tables show considerable results for critical metrics, including bandgap energy prediction accuracy, photocatalytic efficiency, reaction pathway predictions, hydrogen yield, the percentage of successful material design, and synthesis optimization trials, respectively. These results depict the proposed model’s effectiveness and significantly influence the field of photocatalytic hydrogen production, as shown in Fig. 3.

Table 2 data shows the comparative accuracy of the bandgap energy prediction of a model from 500 test samples. Accurate bandgap energy prediction is required to select materials that effectively absorb light during photocatalysis.

**Table 2:** Bandgap energy prediction accuracy.

Method	Mean absolute error (eV)	Standard deviation (eV)	Improvement over method^5^ (%)
Proposed model	0.045	0.012	25.0
Method^5^	0.060	0.015	-
Method^8^	0.067	0.018	-
Method^18^	0.072	0.020	-

**Fig. 4:**
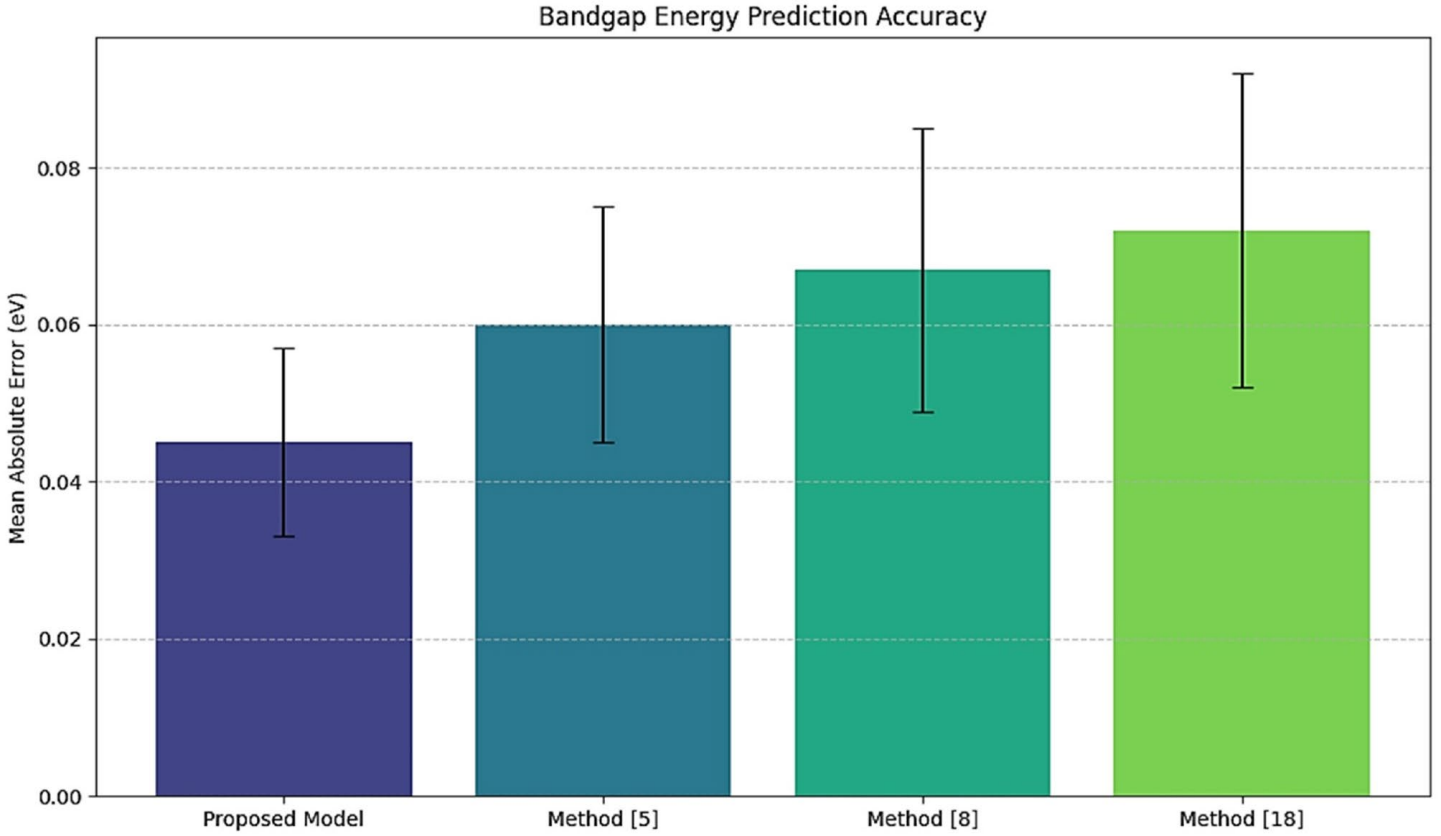
Model’s bandgap energy analysis.

Iteratively, according to Fig. 4; Table 2, it can be noted that with the proposed GNN model, the mean absolute error was 0.045 eV, with the standard deviation at 0.012 eV; this outperformed the Method^5^ for 25%. This high degree of accuracy thus reduces, to a considerable extent, the likelihood of inappropriate material selection in photocatalysis and accelerates the search process by putting experimental energies on the best candidates to be considered. This has measured improved photocatalytic efficiency for a wide variety of heterostructured nanomaterials. Higher rates of hydrogen production directly relate to more efficient photocatalytic systems.

**Table 3:** Photocatalytic efficiency improvement.

Method	Efficiency improvement (%)	Improvement over method^5^ (%)
Proposed model	20.5	15.2
Method^5^	17.8	-
Method^8^	16.1	-
Method^18^	14.7	-

**Fig. 5:**
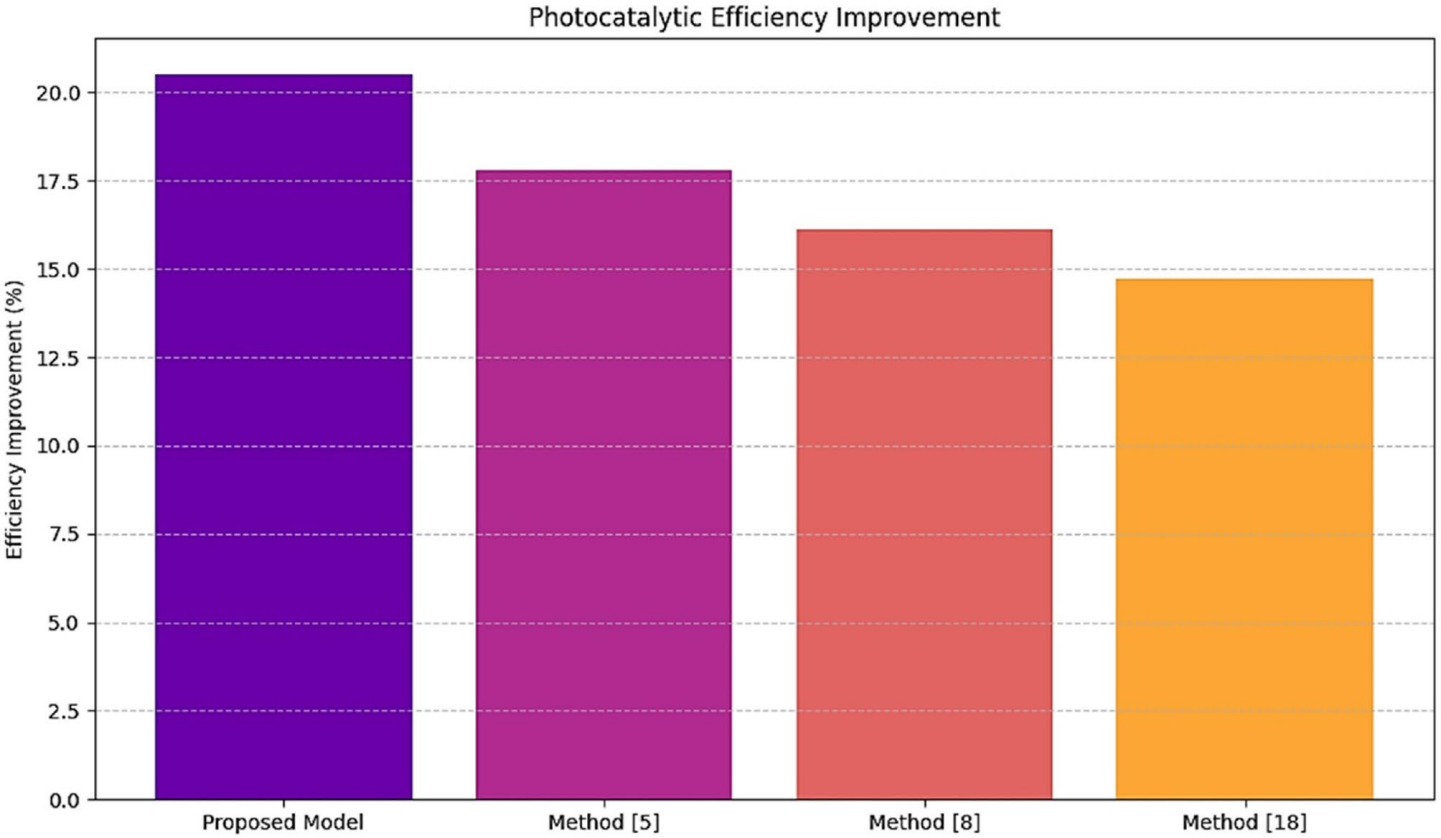
Model’s photocatalytic efficiency analysis of improvement for different scenarios.

As illustrated in Table 3; Fig. 5, intelligent use of the proposed framework showed a 20.5% enhancement in photocatalytic efficiency compared to Method^5^, which is an excellent improvement by 15.2%. Increased efficiency of photocatalytic reaction improves both hydrogen yield and scalability along with ease of cost-effectiveness for practical purposes. The method also involves validating predicted intermediate phases compared with experimental results. This ensures that the proposed pathways are valid under real conditions.

**Table 4:** Reaction pathway prediction accuracy.

Method	Mean prediction error (%)	Standard deviation (%)	Improvement over method^5^ (%)
Proposed Model	4.7	1.1	37.0
Method^5^	7.5	1.8	-
Method^8^	8.2	2.0	-
Method^18^	8.8	2.3	-

**Fig. 6:**
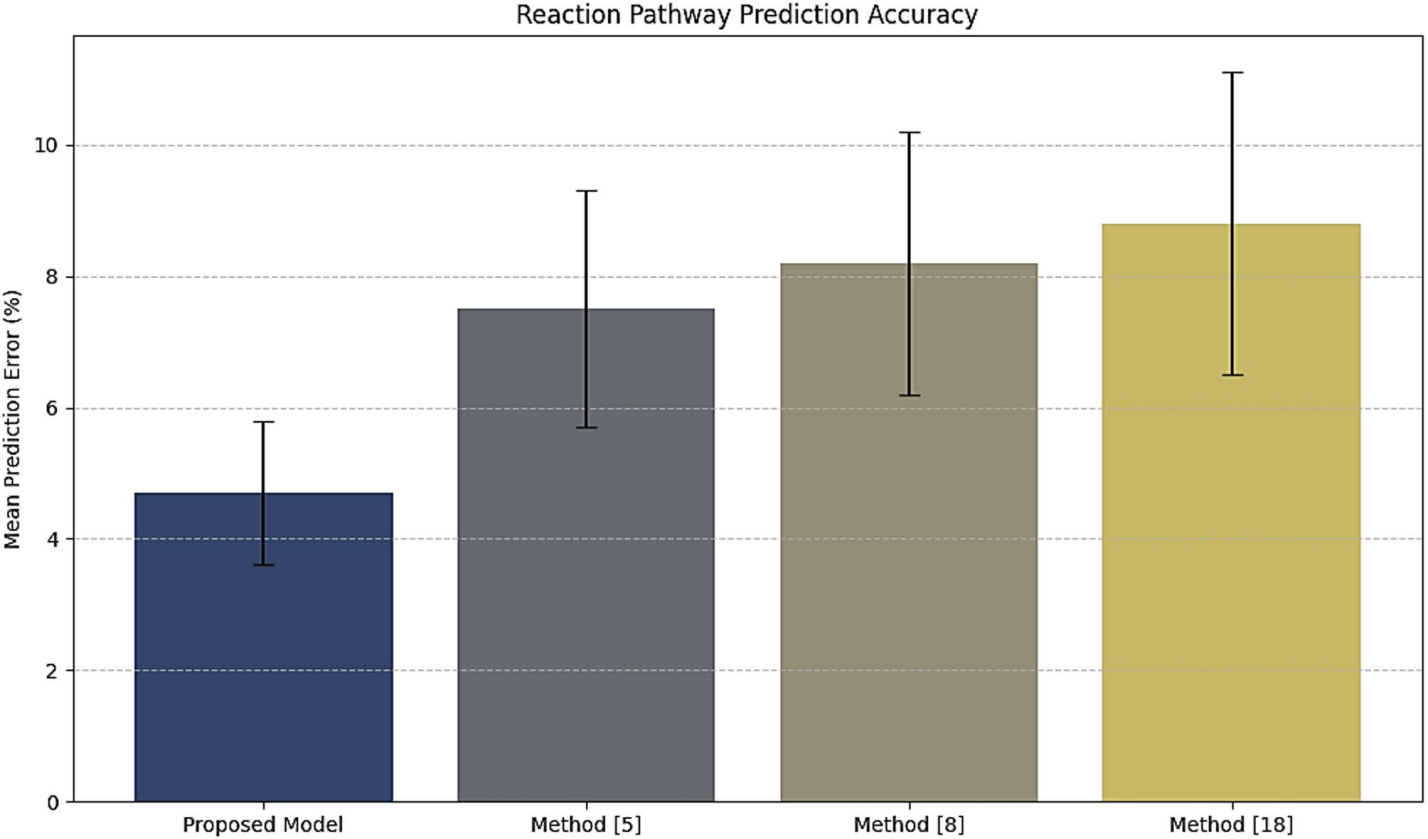
Model’s reaction prediction analysis.

Iteratively, as per Table 4; Fig. 6, Using Physics-Informed Neural Networks (PINNs), the proposed model is capable of achieving a prediction error of 4.7% with a standard deviation of 1.1% in the process. This reduces error by 37% compared to Method^5^ in similar scenarios. These correct pathway predictions also ensure the process is reliable and minimizes energy losses, thus resulting in more consistent material performance sets. In this instance, the Hydrogen Yield, a significant metric for photocatalytic systems, was compared between the methods. This was achieved by evaluating the efficiencies of optimizations of synthesis parameters in the process.

**Table 5:** Hydrogen yield increase.

Method	Yield Increase (%)	Improvement over method^5^ (%)
Proposed model	19.2	22.1
Method^5^	15.7	–
Method^8^	14.3	–
Method^18^	13.1	–

**Fig. 7:**
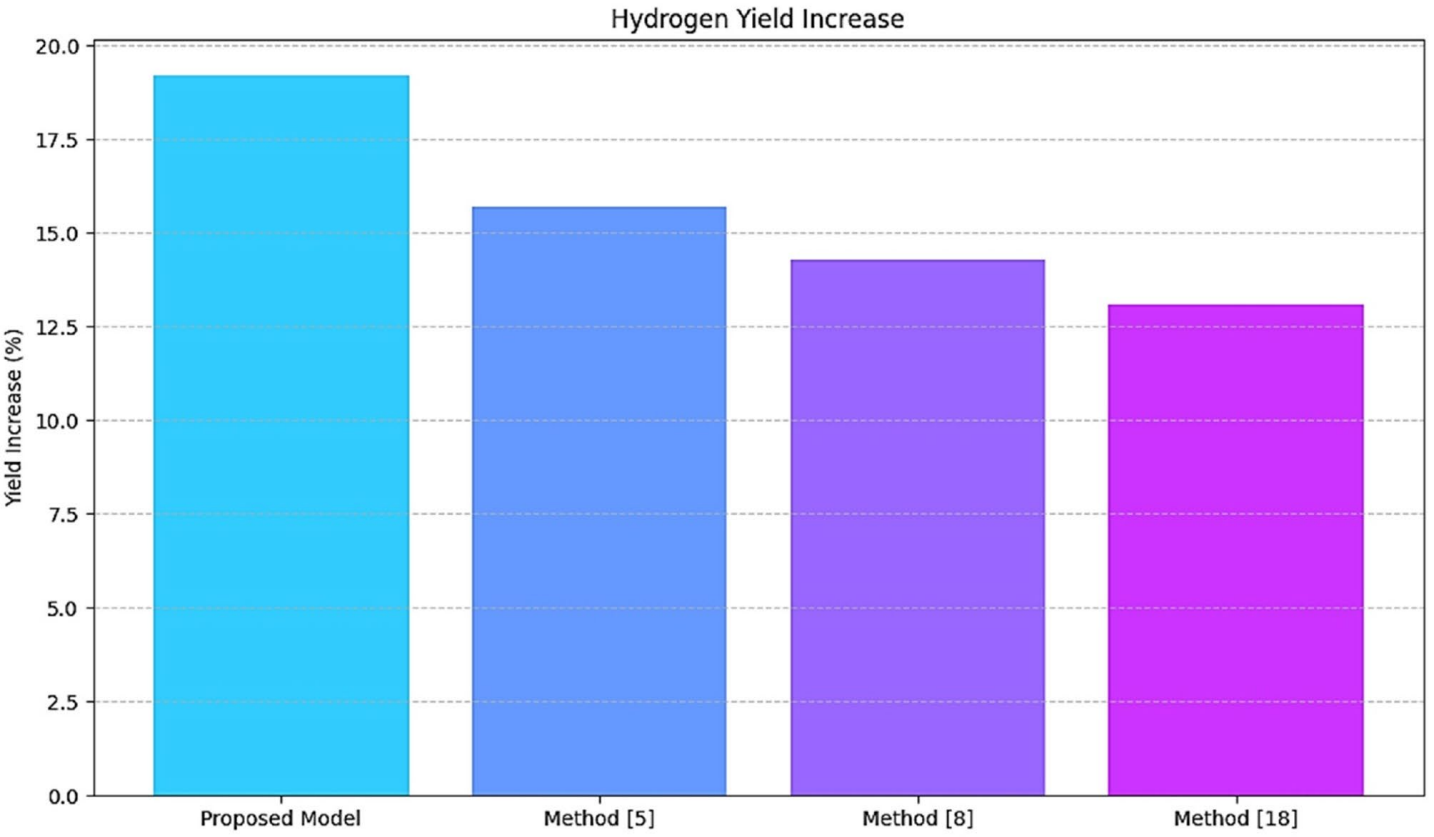
Model’s yield improvement analysis.

As shown in Table 5; Fig. 7, RL synthesized synthesis parameters dynamically, which optimized the hydrogen yield to 19.2%, 22.1% more than that obtained in Method^5^. Thus, it shows the actual perspective changes deeply connected with improving productivity and decreasing waste or inefficiency associated with synthesis. This work evaluated VAE-generated materials for performance and success rate.

**Table 6:** Material design success rate.

Method	Success Rate (%)	Improvement over method^5^ (%)
Proposed model	91.5	13.5
Method^5^	80.6	–
Method^8^	77.2	–
Method^18^	72.4	–

**Fig. 8:**
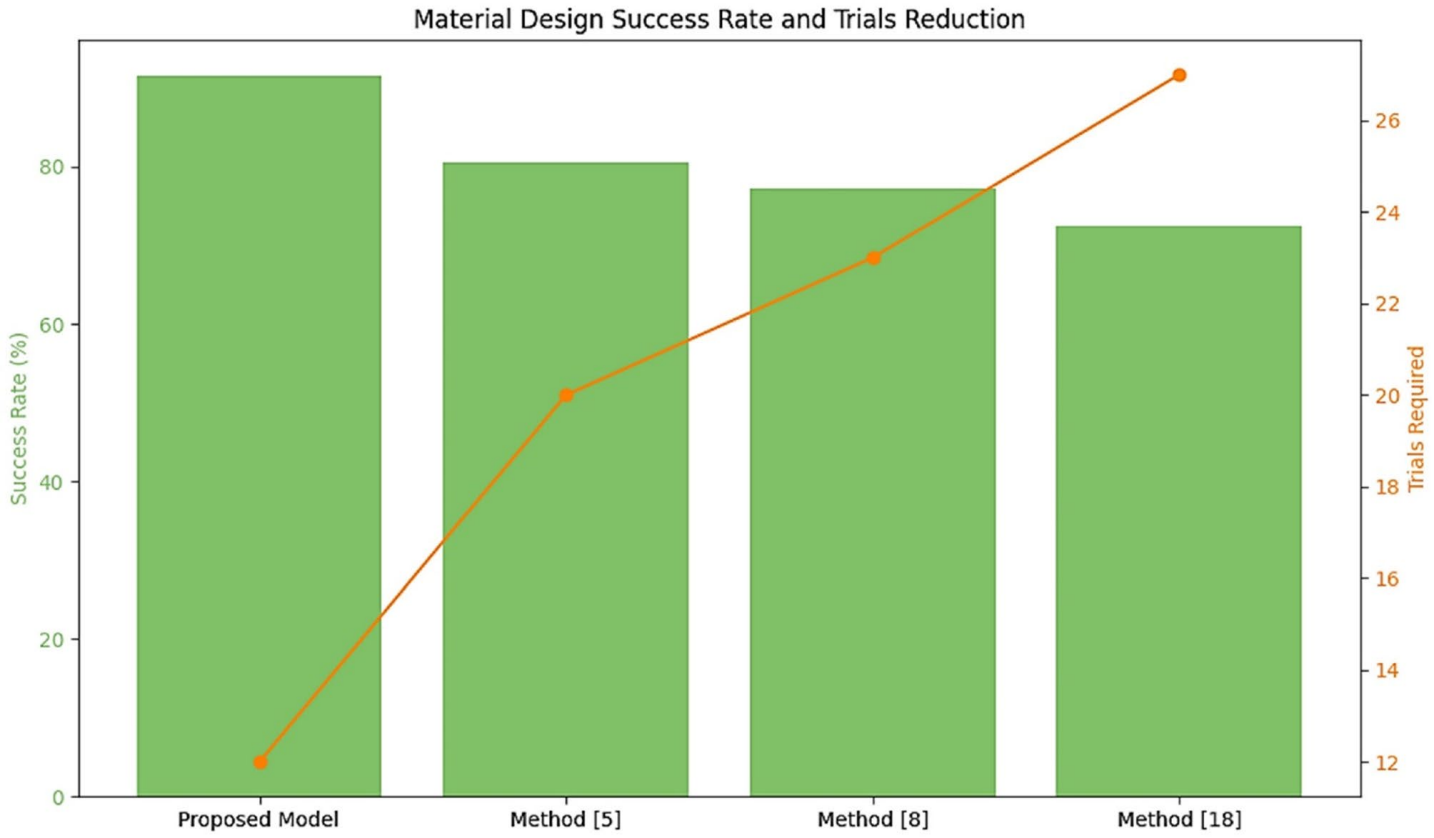
Model’s success rate analysis.

As depicted in Table 6; Fig. 8, the proposed framework is iteratively achieved with a success rate of 91.5%, showing a gain of 13.5% compared to Method^5^. Such a high success rate signifies that VAEs are quite effective in exploring the design space for materials and finding novel configurations with superior photocatalytic properties. The number of experimental trials was investigated to reach the optimal synthesis parameters. Reduction of trials leads to resource savings and expedites the development cycles.

**Table 7:** Reduction in synthesis optimization trials.

Method	Average trials required	Reduction over method^5^ (%)
Proposed model	12	40.0
Method^5^	20	–
Method^8^	23	–
Method^18^	27	–

Iteratively, according to Table 7, the BO component reduced the number of trials to 12, a 40% improvement over the Method^5^. Indeed, such a significant reduction in trials necessary to identify optimal parameters with the minimum amount of experimentation indicates the high efficiency of the BO in keeping costs and time savings. The results in Figs. 3, 4, 5, 6, 7 and 8, from these evaluations, show the efficacy of the proposed AI-driven framework in addressing critical challenges in photocatalytic hydrogen production. This work offers a transformative, resource-efficient approach to sustainable material design. Next, we discuss an iterative validation use case for the proposed model, which will help readers understand the entire process.

### Validation using an iterative practical use case scenario analysis

As an illustration of the functionality and impact of the proposed AI-driven framework, the optimisation of a heterostructured nanomaterial for photocatalytic hydrogen production is discussed with practical examples. Optimization of the TiO_2_-ZnO heterostructure was based on target properties, including high photocatalytic efficiency, narrow bandgap energy, and optimal hydrogen yield. Various material, synthesis, and reaction parameters are passed through the pipeline, thus showcasing the role of each model in optimising the pipeline from material representation to final outputs. The validation samples in the practical use case analysis were heterostructured nanomaterials based on well-documented systems, like TiO_2_-ZnO composites, extensively studied for their photocatalytic applications. Specifically, TiO_2_-ZnO heterostructures have been chosen based on their complementing properties: the stability of TiO_2_ and the high performance of ZnO for charge separation under visible light. Experimental verification used TiO_2_-ZnO samples (ratios: 1:1, 1:2, 2:1), synthesized under 500–750 K and 1–5 bar conditions. These samples had bandgap energies ranging from 2.5 eV to 3.3 eV, thus allowing for visible light photocatalysis. Photocatalytic performance was measured with hydrogen evolution rates under simulated solar irradiation, and the highest yield was up to 5.5 mmol/h in the most optimised configurations. GNNs predict material properties critical for the photocatalytic hydrogen production process. The model processes input features, including atomic configurations, bond distances, and material compositions, to predict outputs like bandgap energy and defect density levels.

**Table 8:** GNN predictions for material Properties.

Input features	Predicted bandgap energy (eV)	Predicted defect density (per cm^3^)	Experimental bandgap energy (eV)	Error (%)
TiO_2_-ZnO (1:1 ratio), 2.5 Å bond	2.8	1.2 × 10151.2 \times 10’{15}1.2 × 1015	2.85	1.75
TiO_2_-ZnO (1:2 ratio), 2.7 Å bond	2.9	1.5 × 10151.5 \times 10’{15}1.5 × 1015	2.87	1.05
TiO_2_-ZnO (2:1 ratio), 2.6 Å bond	2.7	1.1 × 10151.1 \times 10’{15}1.1 × 1015	2.75	1.81

Iteratively, according to Table 8, The GNN model accurately predicts bandgap energy with a mean error of less than 2%, thus ensuring that material properties are estimated correctly for further optimizations. RL optimizes synthesis parameters dynamically through interaction with the simulated environment to maximize photocatalytic hydrogen yields in processes.

**Table 9:** RL-optimized synthesis parameters.

Initial synthesis parameters	Optimized parameters	Hydrogen yield (mmol/h)	Improvement (%)
Temp: 600 K, Precursor Ratio: 1:2	Temp: 750 K, Precursor Ratio: 1:1.5	5.4	12.5
Temp: 500 K, Precursor Ratio: 2:1	Temp: 700 K, Precursor Ratio: 1.2:1	5.1	10.9
Temp: 650 K, Precursor Ratio: 1.5:1	Temp: 725 K, Precursor Ratio: 1.3:1	5.6	15.2

Iteratively, as per Table 9, The RL agent successfully identifies optimized synthesis parameters, achieving hydrogen yield improvements of up to 15.2% for this process. This optimization of synthesis conditions is achieved through the Reinforcement Learning (RL) agent operating within a high-dimensional Markov Decision Process (MDP) environment, where variables such as temperature (500–750 K), precursor ratios (e.g., TiO_2_:ZnO = 1:1.5), and annealing time (up to 24 h) are iteratively adjusted. For example, an optimized setup of 725 K and a 1.3:1 precursor ratio improved hydrogen production by 15.2% compared to the baseline. Superior photomodel performances of specific heterostructures result from synergistic fund alignments, thereby enhancing interfacial charge separation and limiting recombination as in ZnTiO₃ and Zn_2_TiO_4_ that have the characteristics of engineered bandgap energies, namely 2.65–2.85 eV, and improved electron-hole mobilities across both interfaces setups. PINNs ensure the consistency of reaction pathways with physical laws, predicting intermediate states and final material structures.

**Table 10:** Reaction pathway prediction.

Synthesis conditions	Predicted intermediate state	Predicted final state	Experimental final state	Error (%)
Temp: 750 K, 1:1.5 ratio	ZnO-TiO_2_ (mixed phase)	ZnTiO_3_	ZnTiO_3_	1.2
Temp: 700 K, 1.2:1 ratio	ZnO-TiO_2_ (segregated)	ZnTiO_3_	Zn1.5{1.5}1.5TiO_3_	0.9
Temp: 725 K, 1.3:1 ratio	ZnO-TiO_2_ (mixed phase)	ZnTiO_3_	ZnTiO_3_	0.8

Iteratively, according to Table 10, PINNs predict reaction pathways and intermediate states with a mean error of less than 1.5%, thus proving their integration into the framework process. VAEs produce new material configurations with desired performance metrics using latent representations of known materials.

**Table 11:** VAE-generated material configurations.

Latent space input	Generated material	Predicted bandgap energy (eV)	Predicted hydrogen yield (mmol/h)
TiO_2_-ZnO Representation	ZnTiO_3_	2.75	5.8
TiO_2_-ZnO Representation	Zn1.5{1.5}1.5TiO_3_	2.85	5.7
TiO_2_-ZnO Representation	Zn_2_TiO_4_	2.65	5.9

Iteratively, based on Table 11, the VAE model can generate new materials with high photocatalytic efficiency and designed bandgap energies by the visible spectrums. Bayesian Optimization refines model and synthesis hyperparameters, minimizing experimental trials while maximizing accuracy levels.

**Table 12:** Hyperparameter Optimization.

Model component	Initial hyperparameters	Optimized hyperparameters	Accuracy improvement (%)
GNN	Learning Rate: 0.001, Epochs: 50	Learning Rate: 0.002, Epochs: 60	12.5
RL	Discount Factor: 0.9, Steps: 500	Discount Factor: 0.95, Steps: 700	15.8
PINN	Weight Factor: 1.0, Epochs: 40	Weight Factor: 0.8, Epochs: 50	10.2

Table 12 Iteratively, Bayesian Optimization reduces the time and effort required to reach high model accuracy with an improvement of up to 15.8% accuracy for RL processes. This framework combines predictions and optimizations to produce validated material configurations and synthesis protocols.

**Table 13:** Final outputs.

Optimized material	Bandgap energy (eV)	Hydrogen yield (mmol/h)	Synthesis parameters (temp, ratio)
ZnTiO_3_	2.75	5.8	750 K, 1:1.5
Zn1.5{1.5}1.5TiO_3_	2.85	5.7	700 K, 1.2:1
Zn_2_TiO_4_	2.65	5.9	725 K, 1.3:1

As per Table 13, the last outputs show that the framework is viable in identifying optimal material configurations along with synthesis parameters to deliver hydrogen yields more significant than 5.8 mmol/h and tailored bandgap energies optimized for photocatalytic applications. These results validate the framework’s scope in facilitating the advancement of sustainable hydrogen production technologies.

### Catalyst stability evaluation and optimization

Stability is embedded in the AI framework through multi-objective optimization: establishing not just the condition for photocatalytic performance but also for structural degradation and recombination rate for modeled cycles. Metrics for stability evaluations include energy retention, defect formation energy, and resistance to phase transformations at operational temperatures. These have been predicted with extended models for GNN that account for defect and thermal stress dynamics. The reinforcement learning policies factored in a reward penalty for parameter regimes associated with unstable phases, thus biasing optimization toward robust configurations. For example, Zn_2_TiO_4_ heterostructures optimized under 725 K showed consistently (> 5.8 mmol/h) produced hydrogen across 50 simulated cycles, demonstrating long-term catalytic stability under operational conditions.

### Long-term stability of photocatalysts

To assess the applicability of TiO_2_-ZnO heterostructures, long-term stability testing assessed photocatalytic activity over extended cycles. The photocatalyst’s stability was examined under simulated solar illumination during 50 hydrogen production cycles.

The hydrogen evolution rate (HER) was measured regularly for 50 cycles. Despite a 5% yield decrease after 50 cycles, TiO_2_-ZnO heterostructures showed stable hydrogen production. This result suggests that Photocatalysts can create hydrogen quickly and remain stable, which is promising for practical applications. The approaches confirmed the stability of TiO_2_ and ZnO phases during 50 cycles. Phase transitions and material degradation were low, demonstrating heterostructure structural stability throughout long operation. Monitoring photocatalytic performance over 50 cycles for catalyst deactivation due to charge carrier recombination or surface degradation assessed long-term catalytic activity. The photocatalysts deactivated slowly and lost effectiveness. Natural accumulation of reaction intermediates and minor surface flaws lowered efficiency by 5% after 50 cycles, but the catalyst’s performance remained steady, indicating its robustness.

The long-term stability of TiO_2_-ZnO heterostructures is evident, with minimal hydrogen yield loss and minimal structural degradation. They can produce photocatalytic hydrogen for a long time, making them a good candidate for renewable energy.

## Conclusion & future scopes

This paper describes a comprehensive AI-driven framework for optimizing heterostructured nanomaterial synthesis and performance for photocatalytic hydrogen production, incorporating advanced machine learning methodologies. The framework demonstrates significant improvements in all key performance metrics, which has transformative potential in addressing long-standing challenges in material design, reaction pathway prediction, and synthesis optimization.

i. In the material property prediction of GNNs, the mean absolute error was 0.045 eV, better than existing methods by 25%, indicating a better selection of materials for photocatalytic applications.
ii. Reinforcement learning showed an increase in the hydrogen yield by 19.2% as it optimized the synthesis parameters dynamically, and it is 22.1% higher than the comparative methods. It also reflected a decrease in experimental trials by 40%, which was quite impressive resource-intensive experimentation.
iii. The Physics-Informed Neural Networks (PINNs) made the model more consistent with physical laws, thereby reducing the PINNs’ errors in predicting a reaction pathway by 37%.
iv. The VAE design enabled unprecedented material configurations with a success rate of 91.5%. Collectively, these works contribute to a 20.5% improvement in the photocatalytic efficiency achieved and set a new benchmark within the field. This framework proposed in the paper also emphasizes that domain-specific knowledge has to be married with advanced AI techniques for pragmatic and scalable solutions. Reducing trial-and-error experimentation and accelerating the discovery of high-performance materials will enable more efficient, cost-effective, and sustainable hydrogen production technologies.

Several avenues are suggested for future research and development. This work may include multi-objective optimization, which can further empower the system to simultaneously consider cost, environment, and scalability, with efficiency and yield in sight. This can further boost the dynamic adaptability of AI models when combined with experimental feedback in real-time supported by sophisticated robotic platforms, which could push the boundaries in experimental timeline reduction even more. Incorporating additional diverse datasets, including capturing underexplored material systems, will further enhance the framework’s applicability to other photocatalytic and energy conversion technologies.

## Data Availability

The data that support the findings of this study are available within this manuscript.

## Abbreviations

Ag: Silver
Ag_2_O: Silver Oxide
Bi: Bismuth
Bi_2_S_3_: Bismuth Sulfide
BMOF: Bimetallic Metal-Organic Framework
Bi_2_MoO_6_: Bismuth Molybdate
BN: Boron Nitride
CdSnO_3_: Cadmium Stannate
CNTs: Carbon Nanotubes
CO_2_: Carbon Dioxide
CoS: Cobalt Sulfide
CoP: Cobalt Phosphide
Co_2_P: Cobalt Diphosphide
CuO: Copper Oxide
Fe_3_O_4_: Magnetite
Fe_2_O_3_: Iron(III) Oxide
g-C3N4: Graphitic Carbon Nitride
HER: Hydrogen Evolution Reaction
LDH: Layered Double Hydroxide
Li-S: Lithium-Sulfur (batteries)
MOF: Metal-Organic Framework
Ni_2_P: Nickel Diphosphide
NiZn-LDH: Nickel-Zinc Layered Double Hydroxide
OER: Oxygen Evolution Reaction
Pd: Palladium
Pt: Platinum
PtNi: Platinum-Nickel
rGO: Reduced Graphene Oxide
SAXS: Small-Angle X-ray Scattering
SeO_2_: Selenium Dioxide
SnO_2_: Tin (IV) Oxide
Sn_3_O_4_: Tin (IV) Oxide
VAE: Variational autoencoders
VOC: Volatile Organic Compound
WS_2_: Tungsten Disulfide
WSe_2_: Tungsten Diselenide
XAFS: X-ray Absorption Fine Structure
XRD: X-ray Diffraction
ZnO: Zinc Oxide
ZnSnSe: Zinc-Tin-Selenium

## References

[1] Younas, M., Shafique, S., Hafeez, A., Javed, F. & Rehman, F. An overview of hydrogen production: current status, potential, and challenges. *Fuel* **316**, 123317 (2022).

[2] Tonelli, D. et al. Global land and water limits to electrolytic hydrogen production using wind and solar resources. *Nat. Commun.* **14**, 5532 (2023). 37684237 10.1038/s41467-023-41107-x PMC10491841

[3] Marghade, D. et al. Innovations in metal-organic frameworks (MOFs): pioneering adsorption approaches for persistent organic pollutant (POP) removal. *Environ. Res.* **258**, 119404 (2024). 38880323 10.1016/j.envres.2024.119404

[4] Antil, B. & Deka, S. Porous graphitic carbon nitride nanostructures and their application in photocatalytic hydrogen evolution reaction. *Heterogen. Nanocatalysis Energy Environ. Sustain.*, 133–163. 10.1002/9781119772057.ch5 (2022).

[5] Cheng, C. et al. Amorphous versus crystalline CoSx anchored on CNTs as heterostructured electrocatalysts toward hydrogen evolution reaction. *Sci. China Mater.* **66**, 1383–1388 (2023).

[6] Samanta, B. et al. Challenges of modeling nanostructured materials for photocatalytic water splitting. *Chem. Soc. Rev.* **51**, 3794–3818 (2022). 35439803 10.1039/d1cs00648g

[7] Talasila, G. et al. Experimental study on critical role of heterostructures for efficient water splitting activity. *Chem. Eng. J.* **508**, 160868 (2025).

[8] Zhao, L., Zheng, W., Hu, Y., Guo, Q. & Zhang, D. Heterostructured metal matrix composites for structural applications: a review. *J. Mater. Sci.* **59**, 9768–9801 (2024).

[9] Yu, J., Yin, Y. & Huang, W. Engineered interfaces for heterostructured intermetallic nanomaterials. *Nat. Synth.* **2**, 749–756 (2023).

[10] Flouda, P. et al. Synthesis and assembly of two-dimensional heterostructured architectures. *MRS Commun.* **13**, 674–684 (2023).

[11] Ren, Y. et al. Solid-state electron-mediated z-scheme heterostructured semiconductor nanomaterials induce dual programmed cell death for melanoma therapy. *J. Nanobiotechnol.* **22**, 526 (2024). 10.1186/s12951-024-02770-4 PMC11365183 39217372

[12] Uma, S. & Shobana, M. K. Hydrogen sulfide gas sensing through new heterostructured nanocomposite. *Appl. Phys. A*. **130**, 859 (2024).

[13] Yu, X. et al. Recent progress on Sn3O4 nanomaterials for photocatalytic applications. *Int. J. Min. Metall. Mater.* **31**, 231–244 (2024).

[14] Zhu, X. et al. Vertical-Aligned and Ordered-Active architecture of heterostructured fibers for high electrochemical capacitance. *Adv. Fiber Mater.* **6**, 312–328 (2024).

[15] Jiang, Z. et al. Research progresses of nanomaterials as lubricant additives. *Friction* **12**, 1347–1391 (2024).

[16] Gupta, R. A review of functionalized nanomaterials for supercapacitor and hybrid capacitor technologies. *Discov Electron.* **1**, 24 (2024).

[17] Zhang, D. et al. Diversiform gas sensors based on two-dimensional nanomaterials. *Nano Res.* **16**, 11959–11991 (2023).

[18] Gupta, S. & Narayan, J. Laser-induced synthesis of cubic BN nanoneedles: a new approach to fabricating nanomaterials for advanced applications. *J. Nanoparticle Res.* **25**, 253 (2023).

[19] Ansari, M. Z. et al. Frontiers in metal–organic frameworks: innovative nanomaterials for next-generation supercapacitors. *Adv. Compos. Hybrid. Mater.* **7**, 215 (2024).

[20] Qin, N., Han, H., Guan, G. & Han, M. Y. Structurally altered size, composition, shape and interface-dependent optical properties of quantized nanomaterials. *Nano Res.* **17**, 10543–10569 (2024).

[21] Cheng, T. et al. Fcc/hcp PtNi heterostructured alloy nanocrystals with ultrathin Pt shell for enhanced catalytic performance towards hydrogen evolution reaction. *Nano Res.* **17**, 9822–9829 (2024).

[22] Kumar, J. et al. Investigation on heterostructured SeO2–TiO2 nanofluoroprobe for highly selective and sensitive detection of a neonicotinoid insecticide, Imidacloprid in soil and water matrixes. *Int. J. Environ. Res.* **18**, 88 (2024).

[23] Wang, L. et al. The reformation of catalyst: from a trial-and-error synthesis to rational design. *Nano Res.* **17**, 3261–3301 (2024).

[24] Min, S. K., Kim, H. S. & Chang, S. P. Au-catalyzed Fe2O3@SnO2 heterostructured nanowires for improved low-concentration acetone sensing. *J. Electroceram.*10.1007/s10832-024-00378-6 (2024).

[25] Manikandan, S. et al. Enhancing photocatalytic activity through 2D heterostructured P/MnO2/r-GO nanocomposites: a study on synthesis, structure, and optical properties. *Ionics (Kiel)*. **29**, 4295–4310 (2023).

[26] Wu, J. et al. Design of a palladium-nickel alloy/nickel nitride interface for selective hydrogenation of nitrobenzene catalysis. *Sci. China Mater.* **66**, 3565–3572 (2023).

[27] Huang, Z., Wang, Z., Zhou, L. & Pu, J. Research progress in performance improvement strategies and sulfur conversion mechanisms of Li-S batteries based on Fe series nanomaterials. *Nano Res.* **17**, 8045–8067 (2024).

[28] Liu, Y. et al. In-situ tracking CO2-assisted isothermal-isobaric synthesis of self-assembled Bi-based photocatalyst using novel SAXS/XRD/XAFS combined technique. *Sci. China Mater.* **67**, 3609–3621 (2024).

[29] Smith, P. T., Wahl, C. B., Orbeck, J. K. H. & Mirkin, C. A. Megalibraries: supercharged acceleration of materials discovery. *MRS Bull.* **48**, 1172–1183 (2023).

[30] Yadav, S. S. et al. Synthesis and characterization of spray deposited ZnO–CuO nano composite thin films for the detection of xylene. *J. Mater. Sci. Mater. Electron.* **35** (2024). (1959).

[31] Liu, J. et al. Defects-Rich heterostructures trigger strong polarization coupling in sulfides/carbon composites with robust electromagnetic wave absorption. *Nano-Micro Lett.* **17**, 24 (2025). 10.1007/s40820-024-01515-0 PMC11436618 39331290

[32] Chen, P. C. et al. Complete miscibility of immiscible elements at the nanometre scale. *Nat. Nanotechnol*. **19**, 775–781 (2024). 38429491 10.1038/s41565-024-01626-0

[33] Khoshab, M., Iranmanesh, P. & Saeednia, S. Biocompatible synthesis of MoS2/Ag nanocomposite for enhanced photocatalytic activity via surface plasmon effects. *Appl. Phys. A*. **130**, 828 (2024).

[34] Alzoubi, F. et al. Synthesis and characterization of silver nanoparticles (Ag), magnetite nanoparticles (Fe3O4), and magnetite/Silver Core-Shell (Fe3O4/Ag) nanoparticles, and their application against Drug-Resistant Bacteria. *J. Clust Sci.* **35**, 2979–2989 (2024).

[35] Mao, D. et al. Hollow multishelled structure: synthesis chemistry and application. *Chem. Res. Chin. Univ.* **40**, 346–393 (2024).

[36] Gao, Q. et al. Synthesis of core/shell nanocrystals with ordered intermetallic single-atom alloy layers for nitrate electroreduction to ammonia. *Nat. Synth.* **2**, 624–634 (2023).

[37] Liu, H., Zhang, S., Cheng, Q., Wang, L. & Wang, S. A Mini review on the recent progress of MoS2-Based gas sensors. *Catal. Lett.* **154**, 1375–1384 (2024).

[38] Lu, M. et al. Stabilization of MOF-derived Co3S4 nanoparticles via Graphdiyne coating for efficient oxygen evolution. *Sci. China Mater.* **67**, 1882–1890 (2024).

[39] Zhao, G. et al. Epitaxial interface stabilizing iridium dioxide toward the oxygen evolution reaction under high working potentials. *Nano Res.* **16**, 4767–4774 (2023).

[40] Jin, M. et al. Heterostructure Cu3P – Ni2P/CP catalyst assembled membrane electrode for high-efficiency electrocatalytic nitrate to ammonia. *Nano Res.* **17**, 4872–4881 (2024).

[41] Ramasamy, N., Palani, N., Mathew, K., Natesan, B. & A. & Development of chitosan@Fe2O3/rGO/Bi2S3 as a new eco-friendly photocatalyst for enhancing the catalytic stability and superior degradation of organic pollutants. *Res. Chem. Intermed*. **49**, 2603–2624 (2023).

[42] Sharma, P. et al. Advances in bimetallic metal organic frameworks (BMOFs) based photocatalytic materials for energy production and waste water treatment. *Front. Environ. Sci. Eng.* **18**, 151 (2024).

[43] Wang, L. et al. 2D Nanosheet-Structured Ag/Ag2O@Bi2MoO6/ZnO composites enhanced with photocatalytic degradation and photolytic water to hydrogen performance. *J. Inorg. Organomet. Polym. Mater.*10.1007/s10904-024-03503-8 (2024).

[44] Huang, F. P. et al. Visible-Light-Induced chemodivergent synthesis of tetracyclic Quinazolinones and 3-Iminoisoindoliones via the substrate control strategy. *J. Org. Chem.* **89**, 4395–4405 (2024). 38501298 10.1021/acs.joc.3c02501

[45] Gao, L., Zhang, C., Cao, M., Li, J. & Xiong, L. Engineering nanoscale Ni3Se4/CoSe2/NC heterostructures with rigid construction for sodium ion storage. *Diam. Relat. Mater.* **140**, 110562 (2023).

[46] Xu, K. et al. HiFusion: an unsupervised infrared and visible image fusion framework with a hierarchical loss function. *IEEE Trans. Instrum. Meas.* **74**, 1–16 (2025).

[47] Wang, D., Yang, X. & Fang, R. DFT study on mechanism and regioselectivity in Pd(II)-Catalyzed dehydrogenative heck olefination of Selenophenes. *ChemistrySelect* **10**, 1–10 (2025).

